# The ability of artisanal fishers to recognize the dolphins they cooperate with

**DOI:** 10.1186/s13002-020-00383-3

**Published:** 2020-05-29

**Authors:** Daiane S. X. da Rosa, Natalia Hanazaki, Maurício Cantor, Paulo C. Simões-Lopes, Fábio G. Daura-Jorge

**Affiliations:** 1 Departamento de Ecologia e Zoologia, Universidade Federal de Santa Catarina, Florianópolis, Brasil; 2 Department for the Ecology of Animal Societies, Max Planck Institute of Animal Behavior, Radolfzell, Germany; 3 Centre for the Advanced Study of Collective Behaviour, University of Konstanz, Konstanz, Germany

**Keywords:** Individual recognition, Ethnoecology, Traditional knowledge, Fishers’ perceptions, Consensus, Interaction, Cetacean

## Abstract

**Background:**

Human-animal interactions with mutual benefits in the wild are rare. Such positive interactions seem to require an intricate knowledge from the human side on the animals’ behavior and their habitat. In southern Brazil, dolphins and human net-casting fishers have specialized in a cooperative foraging, in which fishers report being able to identify and name dolphins. Here, we evaluate the consensus in their ability to recognize the individual dolphins they interact with. By investigating the reliability of this recognition process, we assess the pros and cons of relying on the fishers’ traditional knowledge to further understand the behavior and ecology of dolphins at the individual level. We also assess the potential role of traditional knowledge for the maintenance of this unusual interaction.

**Methods:**

We interviewed 38 fishers using a semi-structured questionnaire. During each interview, we evaluate their recognition ability of dolphins by showing high-quality photos of dorsal fins of different dolphins, asking questions about the dolphins’ behavior and traits, and about how fishers recognize each dolphin. We also evaluated information about the fishers. Different indices were used to measure the fishers’ ability to recognize dolphins via photos, and their consensus on individual identification. These indices were modeled as functions of traits of both dolphins and fishers to investigate which ones influence the recognition process.

**Results:**

We found that fishers can primarily recognize dolphins by natural marks in the dorsal fin but there was little consensus in recognition. Fishers also tend to repeat the name of the most “popular” dolphins for different photos, indicating low reliability in individual recognition. We also found that fishers who learned from relatives (vertical learning) how to interact with dolphins tend to be more accurate and have higher consensus in dolphin recognition than those fishers who learned from friends (horizontal learning) or individually.

**Conclusion:**

Artisanal fishers have a deep understanding of the dolphins and the system they are inserted in. However, the lack of consensus in identifying individual dolphins herein reported indicates that using their traditional knowledge to further understand dolphin behavior and ecology at the individual level requires caution. Our study also suggests that the transmission of this tradition from parents to sons can be crucial to preserve such a unique human-animal positive interaction in its original form.

## Background

Historically, humans have trained many animals to help them to find food: examples are dogs, falcons, and cormorants. Several of these species are domesticated or have behavioral traits that could be targeted for artificial selection in a process where both species benefit [1]. Human-animal interactions with mutual benefits in the wild, however, are much rarer. Moreover, the costs and benefits involved for both parties are not always fully understood, being in the agenda of a multidisciplinary debate on the nature of such interactions (for a semiotic perspective on human-animal interactions, see [2]). Among the many examples of human and animal interactions, a fascinating case is the interaction between people in Mozambique and Tanzania and a species of wax-eating bird, called the greater honeyguide (*Indicator indicator*). Human hunters and birds work together to access bees’ nests and honey—honey for hunters, beeswax for birds [3, 4]. Such positive interactions seem to be decisive for the evolution of those involved. For instance, the interactions that humans have kept for millennia with scavengers such as vultures, hyenas, and lions, have been crucial in the evolution and welfare of humankind [5]. Nevertheless, this kind of positive interaction becomes complicated when on the non-human side there is a species of high cognitive and social abilities [6, 7]. Cognition comprises processes such as perception, attention, action, and memory, to cite a few [8]. For a positive interaction such as cooperation to happen, perception and memory can aid tracking reciprocity, allowing individuals to weigh the pros and cons involved [9].

Positive interactions between humans and animals also extend to the marine environment—fishers and cetaceans have been historically reported to interact worldwide. One of the first reports was done by the Roman naturalist Pliny the Elder (A.D. 32–79), who described a cooperative hunt between fishers and dolphins during the mullet season in southern France *circa* 70 A.D. [10]. Fishers used to call dolphins that herded mullet schools into positions where fishnets could be easily placed; dolphins apparently caught some of the fish as well. A similar case was reported for Aborigines and dolphins in Moreton Bay, Australia [11]. There, fishers used to make a peculiar splash in the water in attempt to signalize for dolphins the location of the fish—again, mullets—which were herded toward the shore by the dolphins. For these oldest two cases, dolphins’ species were not identified, but both involved mullets as the shared resource. Dolphins and indigenous people cooperation were also reported among the Imragen people of Mauritania, Africa [12]; as well as for Irrawaddy River dolphins in Burma [13], Amazon River dolphin in Brazil [14], and River dolphins in India and China [15]. Although the behavioral and ecological nature of all these interactions are poorly known, the benefits involved seem to be mutual, requiring that both humans and dolphins synchronize and understand each other’s behavior to access and share the same prey.

More insights into benefits, synchrony, and communication in human-animal interactions come from an intensely studied case of artisanal net-casting fishers and bottlenose dolphins (*Tursiops truncatus gephyreus*) in southern Brazil. In Laguna, there is a resident population of bottlenose dolphins (~ 60 individuals) [16], in which some individuals specialized in foraging with net-casting artisanal fishers [17]. Dolphins herd mullet schools toward a fishers’ barrier on the shore awaiting a “stereotyped” behavioral cue by the dolphins—tail slap, head slap, back presentation, or partial emersion [18]. Such cues are understood by the fishers as the right moment to cast their nets. Fishers benefit from this interaction by catching more fish when the dolphins are present and, supposedly, dolphins accrue similar benefits [17]. For the dolphin population, the interaction has further implications, influencing spatial distribution [19], social behavior [20], acoustic behavior [21], and population dynamics [16].

Although it remains poorly understood how this interaction alters fishers’ behavior, it is clear that fishers perceive and rely on the dolphins’ behavior. They synchronize their actions with the dolphins’ actions [17] and reported at least nine ecosystem services from this interaction, including provision, cultural, and social benefits [22]. Interestingly, fishers also report being able to identify dolphins, classifying them as “good” dolphins—those that often interact with them, and “bad” dolphins—those that do not [23]. Fishers, therefore, seem to have valuable knowledge on dolphins’ life-traits and commonly share anecdotes and curiosities about many ecological and behavioral aspects of dolphins at the individual level. Curiously, fishers attribute celebrities names to dolphins—e.g., football players, movie actors, politicians—demonstrating a high level of familiarity.

A long-term study has been monitoring this dolphin population and their interaction with fishers. Long-term studies on population ecology and behavior, however, are money-hungry, time-demanding, and require a systematic effort [24]. Long-term studies are particularly challenging when involving cetaceans, which are wide-ranging, deep-diving, and fast-moving animals [25]. In this context, traditional ecological knowledge can fill some of the gaps of scientific knowledge on the species, habitat, and ecosystems [26–28], enabling quick solutions for co-management and immediate conservation of ecological systems [29–31].

Fishers’ knowledge of cetaceans usually focuses on the general characteristics of the animals (e.g., [32, 33]). The intricate knowledge fishers seem to have on dolphins in Laguna is then an opportunity to understand how the local knowledge about these animals is built individually. However, the fishing community that interacts with dolphins is very heterogeneous, economically, and socially, as well as in their historical engagement in the interactive tactic [22, 23]. For instance, fishers vary in terms of experience in foraging with dolphins. Fishers also fit into different socio-economic profiles: (a) the professionals—those who rely on the fishing activity as the main source of income; (b) the opportunists—those who tend to interact with dolphins only during the mullet season; (c) and the amateurs—those who engage in the fishing with dolphins all year long, but as a hobby, since their main source of income depends on other activities [22]. The analysis of traditional knowledge needs to account for such variability that likely affects the quality and accuracy of individual perceptions on their systems [22, 28, 34].

Here, we investigate the consensus in individual fishers’ perceptions of individual recognition and naming of dolphins they interact with. If fishers are highly consistent in identifying dolphins, their knowledge could be used to describe individual dolphins’ life-traits, such as sex, behavior, age, and kinship relationships—all of which valuable information that requires long-term, systematic sampling effort to be estimated in the wild. We also investigated the factors that can influence this recognition process. By exploring how fishers can recognize dolphins, we have elements to discuss: (a) to which extent the detailed fishers’ knowledge can be used to complement ecological information on dolphins; (b) how intricate the dolphin-fisher foraging interaction is; (c) and how critical the fishers’ traditions and perceptions of the dolphins’ lives might be for this interaction to persist—a possible requisite for all positive dolphin-human interactions.

## Methods

### Research area and data collection

**Fig. 1:**
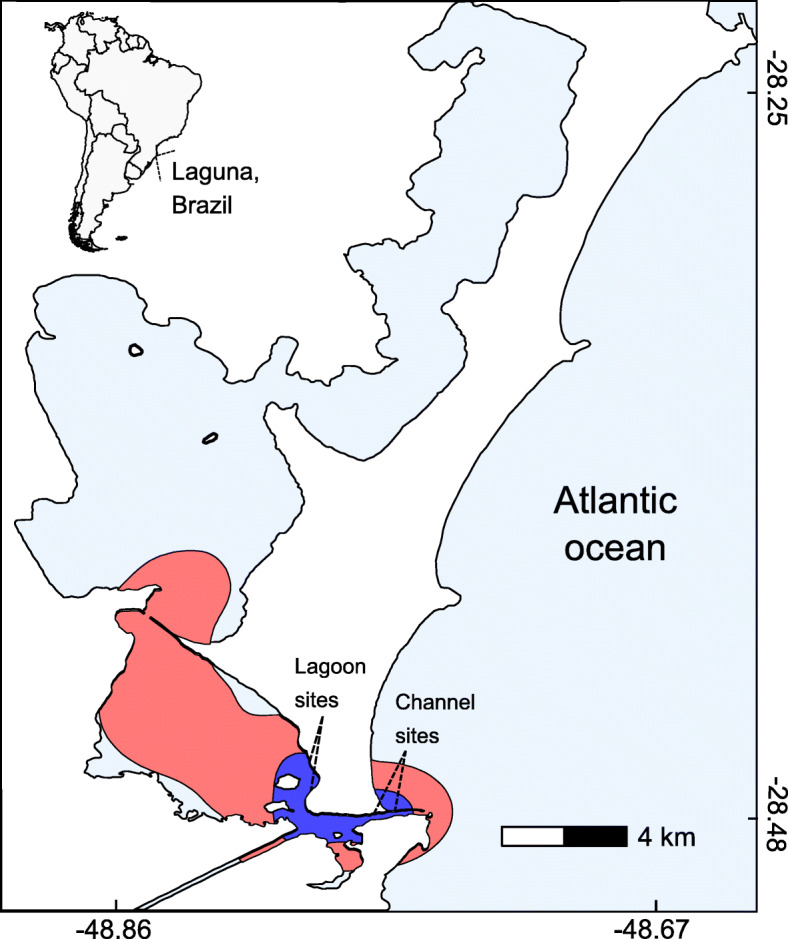
The research area: The lagoon system in Laguna, southern Brazil. The red and blue areas indicate, respectively, the estimated home ranges of dolphins [16, 19] that tend to forage independently (“bad dolphins”) and those that tend to interact with fishers (“good dolphins”). Interviews occurred at the fishing sites indicated

We interviewed fishers between September 2008 and August 2011 around the lagoon system of Santo Antônio dos Anjos, adjacent to the city of Laguna, southern Brazil (28°20′ S and 48°50′ W; Fig. 1). Dolphin-fisher foraging interactions frequently occur in at least five fishing sites close to the Tubarão River and the inlet channel (details in [22]). Interviews were conducted around four of these five fishing sites, when fishers were waiting for dolphins to interact (*Tesoura* and *Toca da Bruxa* across the inlet channel, *Ponta das Pedras* and *Barra* inside the lagoon; Fig. 1) or at fishers’ houses. Prior to each interview, participants were asked to sign a free informed consent and data release form.

Participants were interviewed individually following a semi-structured interview protocol. The interviews were digitally recorded and contained questions about the fishers’ characteristics, such as age—a proxy for fishing experience—, the fishing site where they frequently fish with dolphins; how they learned to fish with dolphins; and if fishing with dolphins was their primary activity. We also presented a photo-identification catalog of 30 dolphins containing high-quality photos of dorsal fins and natural marks. The catalog was elaborated following well-known photo-identification protocols [35] as part of a systematic survey program to monitor the dolphin population (for details on the survey methodology, see [16, 36]). For each interviewee, we first presented 8–10 photos of different dolphins chosen at random, to avoid biases in the amount of information recorded per fisher. Then, for each photo, we asked questions about the dolphins’ individual traits (name, sex, age, and if it was a “good” or “bad” dolphin), and how the fishers recognize each dolphin (e.g., if by marks on the dorsal fin or other characteristics, such as behavior, or body shape).

### Data analysis

The individual dolphin identification by fishers comprises three processes: the recognition of the individual dolphin in a given photo; the attribution of a name to that dolphin; and the association of that dolphin to a set of traits. These processes can be influenced both by dolphins’ and fishers’ characteristics. To account for these potential influences, we used the characteristics of dolphins (see below) estimated from a long-term monitoring effort in the area [36, 37] and the characteristics of fishers collected in the interviews to test which, if any, affect the ability of fishers in recognizing dolphins. We then quantified the following: (1) how many times each photo was recognized; (2) the degree of agreement in naming dolphins through photos; (3) the degree of consensus among fishers in the dolphins’ recognition and naming; (4) the characteristics of the dolphins and the fishers that influence recognition and consensus, and; (5) the recurrence of names attributed to dolphins.

### Recognition and concordance in photo identification

To quantify how many times each dolphin photo was recognized, we calculated the Recognition Index (RI) as the number of positive recognitions of a given photo by the total number of presentations of this photo to all fishers. To quantify the degree of agreement in recognition of each photo, we calculated the Concordance Index (CDI) (adapted from [38]) as: (*nR* − *nN*)/( *nR* − 1), where *nR* is the number of times a photo was recognized, and *nN* is the number of different dolphin names assigned to each photo. Both RI and CDI range between 0 and 1, and we assumed a cut-off of 0.5 to define high degrees of recognition and concordance.

To model both indices as a function of dolphins’ characteristics, we fitted a beta generalized linear model (GLM), with a logit link function [39]. The explanatory variables were the characteristics of the dolphin in the photo and how they were recognized by the fishers. We used three characteristics estimated during the long-term, systematic survey program in the area: home range (kernel densities based on photo-identification; see [36]); encounter rate (resighting of photo-identified individuals over time; estimated in [37]); predominant foraging behavior classified in “good” or “bad” dolphin (classified in based on the frequency of foraging with fishers *versus* foraging independently; see [19]). The variables on dolphin recognition by fishers were form (whether the dolphin was recognized by either its body shape or general behavior) and marks (whether the dolphin was recognized by the presence of scratches, epidermal lesions, and/or long-lasting marks in their dorsal fin).

### Consensus among fishers

To assess the degree of consensus among fishers in recognition of dolphins, we used the Consensus Index (CI) as the number of matches in recognized photos (the same name given to the dolphin) by the number of similar photos presented for each pair of fishers. We restricted this analysis to 26 out of the 38 interviewees to whom we presented at least the same three photos to reduce the bias of small samples. The CI ranges between 0 (no consensus) and 1 (total consensus) between a pair of fishers. To obtain a consensus measure per fisher, we calculated the Average Consensus Index (ACI) as the average of all CIs of a given interviewee with all other pairs of fishers (adapted from [38]). We fitted a beta generalized linear model (GLM), with a logit link function [39], to model ACI as a function of the following fishers’ characteristics: age, the main fishing site—given that the lagoon sites are used by more professional fishers, while the channel sites also by opportunists and amateurs fishers [22]—, whom they learned how to fish with dolphins (whether by themselves, from other unrelated fishers, or from relatives), and whether fishing with dolphins was their main activity or not.

### Certainty on naming dolphins

Some of the names given to individual dolphins by the fishers were more common than others, which means that they tend to be given to more than one photo. To measure this potential error and to investigate what can favor this inaccuracy, we calculated a Certainty Index (CeI) as the number of times a name was assigned by fishers to the same photo divided by the number of times that name was cited for all photos presented during all interviews. Some names are more popular than others and tend to be cited more often. To account for this popularity bias, we corrected the Certainty Index (CeIc) by multiplying it by a correction factor (CF)—the number of times a name was cited divided by the number of citations of the most popular name (adapted from [40]). For each dolphin name, fishers also reported the perceived age, sex, behavior (i.e., whether the dolphin was “good” or “bad” dolphin), and the recognition method (i.e., whether through dolphin’s body form or marks; see above). As the same name was cited several times by different fishers, we considered the most frequently cited attribute for that name and the average perceived age. We then modeled the CeIc as a function of these characteristics coupled to dolphin names using a beta GLM, with a logit link function [39].

### Model selection

All models considered additive and isolated relationships between the response and the explanatory variables—interactive terms were not implemented due to overparameterization issues. We started from the full models and selected variables by stepwise backward elimination, using Akaike’s information criterion (AIC) and Akaike weight to rank and find the most parsimonious model by favoring the one with the lowest AIC. We considered fitted models the ones with ΔAIC< 2 [41]. All models and graphs were built and evaluated in R [42], using the packages “glmmTMB” [43] to fit the models, “MuMIn” [44] for calculations of AIC and Akaike weight, and “DHARMa” [45] for scaled residuals checking. The significance level in statistical tests was 95% (*p* < 0.05).

## Results

We interviewed 38 fishers with ages ranging from 18 to 82 years old, among which 22 were least 51 years old. Only six fishers had less than ten years of experience in fishing with dolphins, and 20 of them learned how to fish with their parents. All fishers were male.

### Recognition and concordance of dolphins in photos

Twelve out of the 38 interviewees recognized all dolphins presented in photographs; the remaining recognized at least 1 of the photographed dolphins. Among the 30 photos of dolphins, only 2 were never recognized. The average Recognition Index was RI = 0.63 ± 0.15SD, suggesting a high degree of recognition of the photos presented. However, the Concordance Index suggested that the degree of agreement in the recognition was low (CDI = 0.41 ± 0.32SD). Dolphins on photos #15 (frequently named as *Caroba*), #37 and #33 (both frequently named as *Scooby*) presented the highest *CDI* values (between 82% and 100%), while dolphins on photos #41, #24, and #18 (with unknown or uncertain names) had the lowest *CDI* (between 20% and 1%).

**Fig. 2:**
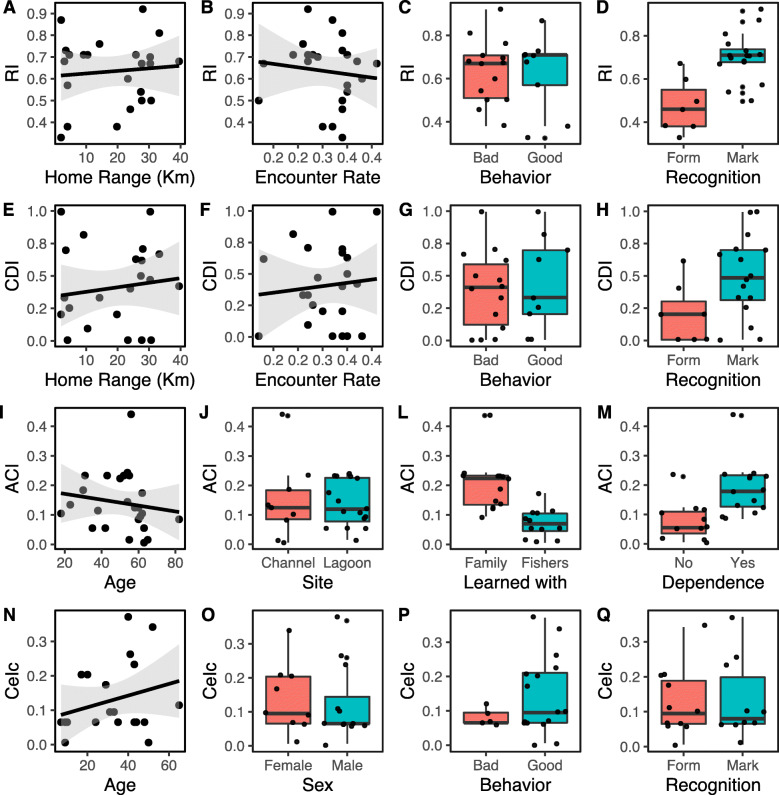
Variation in the ability in recognize individual dolphins, concordance, and consensus among artisanal fishers across a set of explanatory variables. Recognition index (RI) does not change significantly with dolphin home range (**a**), dolphin encounter rate (**b**), dolphin behavior (“good” or “bad” dolphin; **c**); but the index increases when dolphins are recognized by marks in the dorsal fin in comparison to body shape and general behavior (form) (**d**). The Concordance index (CDI) does not change with dolphin home range (**e**) and encounter rate (**f**), dolphin behavior (“good” or “bad” dolphin; **g**); but it increases when dolphins are recognized by marks in the dorsal fin (**h**). The average consensus (ACI) do not vary with fishers’ age (**i**) and preferable fishing site (**j**); it is significantly higher when fishers learn how to fishing with dolphins from parents or other relatives (families) (**l**) and it increases slightly whether they depend on this fishing activity (**m**); the consensus in naming dolphins (*CeI*c) does not change significantly with the perceived age (**n**), sex (**o**), behavior (**p**) or type of recognition (**q**) of the dolphins

The Recognition Index (RI) was practically constant regardless of the dolphins’ home range and encounter rate, suggesting that the presence of dolphins in the area and the frequency they are sighted do not affect their recognition by fishers. There was also no variation in RI whether dolphins tend or not to interact with fishers (i.e., “good” and “bad” dolphins, respectively). Regarding the way dolphins are recognized, unmarked individuals—those that were recognized by body shape or behavior displays—were less recognized than those with long-lasting marks in their dorsal fin. The most parsimonious linear models included recognition type and encounter rate (model RI3, see Additional file 1), but only the recognition by marks in dorsal fin had a significant effect on individual dolphin identification (estimate = 1.065; *z* value = 4.947; *p* < 0.001; Fig. 2).

The Concordance Index (CDI) also did not vary significantly with home range and encounter rate, and there were no differences between “good” and “bad” dolphins. However, unmarked individuals had the lowest CDI, while individuals identified by marks in dorsal fin tended to be recognized with greater concordance (estimate = 1.093; *z* value = 1.977; *p* = 0.048, Fig. 2). The most parsimonious linear model included only the recognition type (model CDI4, Additional file 1), suggesting a low certainty in recognition of unmarked individuals.

### Consensus among fishers

The average consensus per fisher (ACI) ranged from 0 to 0.44 (ACI = 0.13 ± 0.10SD). The fishers’ age, favorite fishing site, and dependence on the interaction with dolphins (whether or not they live exclusively of this activity) do not affect their consensus in individual identification. The only variable that influenced the consensus in identification was with whom fishers learned how to interact with dolphins (estimate = − 1.216; *z* value = 0.249; *p* < 0.001; Fig. 2; model ACI4, Additional file 1). Fishers who learned to fish alone (individual learning) or from unrelated fishers (horizontal learning) tended to misidentify dolphins more often (lower ACI) than those who learned how to fish from their parents or other relatives (vertical learning).

### Certainty on naming dolphins

We found a low degree of certainty (CeI = 0.12 ± 0.10 SD), meaning that different fishers gave the same dolphin name to several different dolphins’ photos. The corrected Certainty Index (CeIc) was not affected by any dolphin covariate (age, sex, behavior, recognition type), suggesting that fishers tend to give a name for a dolphin even when they are not certain, independently of the dolphins’ characteristics (Fig. 2).

## Discussion

Traditional ecological knowledge and perceptions of a natural environment are exposed to systems’ changes, cultural losses, and the relationships between stakeholders and the ecological processes (see [46, 47]). Our findings on the reliability and accuracy of an element of the traditional knowledge of fishers involved in a rare interaction with wild dolphins provide insights on how these fishers perceive this system and how this system works. These insights can further indicate how critical the maintenance of the fishers’ tradition would be for the persistence of this rare interaction that depends on how dolphins and fishers synchronize their behavior. Although the fishers in Laguna can describe and understand many biological aspects of the dolphins’ behavior and perceive the major threats and values related to dolphins and their interaction [22, 23], only now their empirical ability to recognize and name individual dolphins—a refined piece of their knowledge—was tested.

Our findings on the fishers’ degree of recognition and consensus in identifying dolphins—a requisite for the use of knowledge fishers have on each individual dolphins—indicate that although fishers can recognize dolphins, there is a substantial degree of uncertainty and lack of consensus among fishers when naming the individual dolphins. Interestingly, this uncertainty is reduced when fishers learn how to interact with dolphins from their parents or relatives, suggesting that (a) the vertical learning of the fishing tactic with dolphins might be an essential element for fishers to maintain a refined and robust understanding of the system they take part in; (b) one should use data from only those fishers that vertically learned the fishing tactic when the aim is to assess information on individual dolphin level. Further studies, however, should investigate whether this vertical learning interferes with other aspects of fishers’ perception.

Our findings also suggest that fishers tend to recognize dolphins by the dorsal fin. However, they gave different names for the same dolphin and the same name for different photos of dolphins, indicating that disagreement is common. The variation in dolphin recognition could be first a consequence of how often each dolphin interacts with the fishers. Thus, we expected that those dolphins with smaller home ranges—usually those that use areas closest to the interaction sites (e.g. [19])—, or those that are often in the fishing sites, would be more easily recognized. However, only the presence of long-lasting and visible marks on the dolphin dorsal fin increased the chance of recognition and the concordance among fishers. Thus, in essence, the fishers’ process of recognizing dolphins does not differ from the individual recognition procedure performed by systematic photo-identification protocols, commonly applied to small cetaceans, through long-lasting marks in the dorsal fin or body [35]. We cannot state, however, that the recognition of dolphins by fishers is a simple process. It might require additional elements not fully explored here, such as the memorization of individual marks and the ability to discriminate the behaviors displayed by dolphins when interacting with fishers—i.e., the tail slap, head slap, back presentation or partial emersion—which cannot be perceived and recognized via photographs but can be influenced by fisher’s experience and familiarity with the system. Further studies should consider these other potential forms of recognition.

Nevertheless, recognizing individual dolphins may also depend on the fishers’ characteristics. Although age—our proxy for fishing experience—preferred fishing site and economic dependence on the interaction with dolphins did not affect the fishers’ consensus in dolphin recognition, the way they have learned the tactic did. We found that fishers who learned how to interact with dolphins from their relatives (primarily their fathers), even when not very experienced (young fishers), agree more often in the dolphin recognition than those who learned from unrelated fishers. These results suggest an interesting side of this process not previously hypothesized. The tactic of fishing with dolphins is complex and requires a deep understanding of the dolphins’ stereotyped behaviors, as well as the fish schooling behavior, and the proper technique of casting nets [22, 23]. Therefore, those fishers that learned how to recognize dolphins may have better learned other aspects related to the dolphins’ and mullets’ behavior that are crucial for the synchrony of the interaction.

However, mastering all these aspects requires proper training and guidance. The dolphin-fisher system is open for fishers from neighboring communities that occasionally came to fish with dolphins or even the amateurs and opportunists who have this fishing activity as a hobby (see [22]). Those fishers tend to learn how to “play the game” with dolphins by copying the professionals or copying those fishers who are more experienced, more dependent of the system, or have a family history of participation in this tactic. Therefore, learning by copying and observing other fishers, without a closer relationship between learners and mentors, could increase the chances of mistakes, the loss of details, and refinements of the technique—as did with the recognition ability—or even the invasion of innovative but non-adaptive behavioral variants. Indeed, vertical learning—from parents to offspring—may favor the transmission of complex behaviors, without significant innovations or distortions, while horizontal learning may be a less conservative and more propagative process, usually favored in changeable environments’ context [48–51].

The learning process of the fishing tactic—for both dolphins and fishers—seems key for the maintenance and stability of this and all other positive dolphin-human interactions. In fisheries-dependent communities, fishing is a source of identity for the individual, motivating fishers to continue their activity even when benefits are very low [52]. However, in this dolphin-human system in Laguna, finding young fishers who learned the tradition from their relatives has become increasingly rarer in these days and ages. Whether fishers change their behavior—as a consequence of an open learning process caused by copying from others and innovations that might be rapidly transferred among fishers—it could affect the system unpredictably, since dolphins, as well as mullets, respond in a synchronized and ritualized way to the fishers behaviors, and vice-versa [17]. Whether fishers change the way they interact, dolphins may also adapt their behavior. Therefore, maintaining the fisher tradition and how they participate—with their own rules, norms, and regulations (see [23])—seems critical to maintain the sustainability of this system.

We also found that fisher’s certainty on the name of the dolphin was low, as the same name tends to be used to identify different photos. This uncertainty is not dependent on the dolphins’ traits, such as being older, male or female, or often interact with fishers. Some dolphins are more popular than others, and this seems to be related to a combination of factors perhaps not captured by our models—i.e., the dolphin’s individual efficiency in increasing the fishers’ catch. For example, *Caroba*, *Scooby*, and *Figueiredo* are very popular dolphins in the area. Why did fishers often cite them for more than one photo? Certainly, fishers fail in the recognition process; but apparently, fishers also avoid an “I don’t know” response, opting to cite names of well-known and “popular” dolphins and then have more chance of success in the identification.

The fishers’ tendency to answer even when they are unsure of the right response can be an issue for studies based on traditional knowledge, and filters should be done to avoid this bias [28]. While fishers provide invaluable information on dolphins, it seems that part of that—at least those that require dolphin recognition—might not be accurate and reliable. Reliability is the confidence that interviewees are mentioning what they really know and believe, while accuracy is the degree to which the information provided by interviewees, even if reliable, corresponds to real biological phenomena [53]. However, we should also consider that under an emic perspective and in an ethnotaxonomic sense, this lack of accuracy can also be attributed to lumping and splitting errors, which occurs when multiple similar units are grouped under one name, or to the same unit is attributed more than one common name [40]. Here, the little consensus in classifying individual dolphins suggests a lack of accuracy, while the use of “popular” dolphin names as default and safety response suggests a lack of reliability. These findings do not invalidate information offered by fishers—also, as aforementioned, because fishers could recognize dolphins by other mechanisms not explored here—but claim for cautions in the use of the fishers’ knowledge for scientific purposes when an individual recognition is needed.

## Conclusions

We found that fishers can indeed recognize dolphins, but there is little consensus among them. These findings suggest both a lack of reliability and accuracy and so call for caution when using the traditional knowledge for understanding particular elements of this system—such as ecological aspects of dolphins at an individual level—from a scientific perspective. Our results further suggest that the ability of the fishers in recognize dolphins is affected by how their traditional knowledge related to the foraging tactic is transmitted, being more accurate and with a higher consensus when fishers learn the tactic of fishing with dolphins from relatives. Further studies should better investigate whether the vertical transmission is important not only for the ability of fishers in recognizing dolphins but also for the learning of all the nuances related to this rare human-animal interaction. If vertical learning plays an important role, it will be key to investigate the consequences of disrupting this process for the persistence of the tactic. Other positive dolphin-human interactions have become locally extinct worldwide (reviewed in [54]). While these disappearances might have multiple causes, cultural losses of fishers’ traditional knowledge might have played a part in it; indeed, human cultural losses are generally linked with the loss of biodiversity and ecosystem services [55]. Then, we highlight that to conserve this rare dolphins-fishers interaction in its original form, it is likely necessary to protect the fishers’ tradition by encouraging its transmission via vertical learning, from the next generation of fathers to their sons.

## Supplementary information

**Additional file 1.** Model selection table for Recognition Index (RI), Concordance Index (CDI), consensus per fisher (ACI) and degree of certainty (CeI).

## Data Availability

The R code to reproduce the analyses is available in the supplementary material; the data can be made available upon request, without disclosing the identity of interviewees; the dolphin photo-identification catalog used during the interviews is also available upon request.
