# Supplementary material for: The ability of artisanal fishers to recognize the dolphins they cooperate with
Source: J Ethnobiol Ethnomed. 2020 May 29;16:30. doi: 10.1186/s13002-020-00383-3 (PMC7257239; doi:10.1186/s13002-020-00383-3)
Supplement: Supplementary file 1 — Additional file 1. Model selection table for Recognition Index (RI), Concordance Index (CDI), consensus per fisher (ACI) and degree of certainty (CeI). [file 13002_2020_383_MOESM1_ESM.pdf]

ADDITIONAL FILE 1: Model selection table

Title: The ability of artisanal fishers to recognize the dolphins they cooperate with.

Daiane S. X. da Rosa; Natalia Hanazaki; Maurício Cantor; Paulo C. Simões-Lopes; Fábio G. Daura-Jorge*

*corresponding author: daura.jorge@ufsc.br

We fitted beta generalized linear models (GLM), with a logit link function, to model: 1) the Recognition Index (*RI*) and Concordance Index (*CDI*) as a function of dolphins' characteristics; 2) the Average Consensus Index (*ACI*) as a function of fishers' characteristics; 3) the Certainty Index (*CeI*) as a function of characteristics of dolphins named. Model selection procedure used Akaike's information criterion (AIC) and Akaike weight to rank and find the most parsimonious model by favouring the one with the lowest AIC. Table A1 shows the rank and the most parsimonious models for each response variable.

Table A1. Model selection for Recognition Index (RI), Concordance Index (CDI), consensus per fisher (ACI) and degree of certainty (CeI). The estimates for each explanatory variable are presented: encounter rate (ER), behavior (Beh), home range (HR), recognition (Rec), age (Age), dependence (Dep), learning (Ler), site (Site), sex (Sex). Models were calculated considering a beta distribution due the response variable range from 0 to 1. df=degrees of freedom; logLik=log of probability; AIC= Akaike Information Criteria; $\Delta AIC = AIC_{model} - AIC_{minimum.}$ *Significant estimates.

RI Models	Int	ER	Beh	HR	Rec	df	logLik	AICc	ΔAIC	weight
3	0,82	-3,17	.	.	+*	4,00	19,78	-29,33	0,00	0,46
4	-0,10	.	.	.	+	3,00	18,17	-29,08	0,25	0,40
2	0,71	-3,22	.	0,01	+	5,00	20,10	-26,66	2,67	0,12
1	0,76	-3,15	+	0,00	+	6,00	20,11	-22,98	6,35	0,02
5	0,55	.	.	.	.	2,00	11,28	-17,95	11,38	0,00
CDI Models	Int	ER	Beh	HR	Rec	df	logLik	AICc	ΔAIC	weight
4	-1,12	.	.	.	+*	3,00	7,46	-7,65	0,00	0,54
5	-0,36	.	.	.	.	2,00	5,54	-6,48	1,17	0,30

3	-1,22	.	.	0,01	+	4,00	7,50	-4,77	2,88	0,13
2	-1,63	.	+	0,02	+	5,00	7,63	-1,73	5,92	0,03
1	-1,80	0,67	+	0,02	+	6,00	7,64	1,97	9,62	0,00
ACI Models	Int	Age	Dep	Lear	Site	df	logLik	AICc	ΔAIC	weight
4	-1,36	.	.	+*	.	3,00	35,18	-63,22	0,00	0,47
3	-1,81	.	+	+	.	4,00	36,49	-62,97	0,25	0,41
2	-1,60	0,00	+	+	.	5,00	36,63	-60,11	3,11	0,10
1	-1,59	0,00	+	+	+	6,00	36,63	-56,60	6,61	0,02
5	-1,82	.	.	.	.	2,00	26,13	-47,71	15,51	0,00
CeIc Models	Int	Age	Beh	Rec	Sex	df	logLik	AICc	ΔAIC	weight
5	-1,91	.	.	.	.	2,00	22,80	-40,94	0,00	0,65
4	-2,21	0,01	.	.	.	3,00	23,21	-39,00	1,94	0,25
3	-2,77	0,02	+	.	.	4,00	23,72	-36,93	4,00	0,09
2	-2,75	0,02	+	+	.	5,00	23,75	-33,50	7,43	0,02
1	-2,84	0,02	+	+	+	6,00	23,77	-29,53	11,40	0,00
